# HyPer2 imaging reveals temporal and heterogeneous hydrogen peroxide changes in denervated and aged skeletal muscle fibers *in vivo*

**DOI:** 10.1038/s41598-019-51035-w

**Published:** 2019-10-08

**Authors:** C. A. Staunton, E. D. Owen, N. Pollock, A. Vasilaki, R. Barrett-Jolley, A. McArdle, M. J. Jackson

**Affiliations:** MRC-Arthritis Research UK Centre for Integrated research into Musculoskeletal Ageing (CIMA), Department of Musculoskeletal Biology, Institute of Ageing and Chronic Disease, University of Liverpool, Liverpool, L7 8TX UK

**Keywords:** Experimental models of disease, Ageing

## Abstract

To determine the role of denervation and motor unit turnover in the age-related increase in skeletal muscle oxidative stress, the hydrogen peroxide (H_2_O_2_) specific, genetically-encoded, fluorescent *cyto-HyPer2* probe was expressed in mouse anterior tibialis (AT) muscle and compared with *ex vivo* measurements of mitochondrial oxidant generation. Crush of the peroneal nerve induced increased mitochondrial peroxide generation, measured in permeabilised AT fibers *ex vivo* and *intra vital* confocal microscopy of *cyto-HyPer2* fluorescence showed increased cytosolic H_2_O_2_ in a sub-set (~24%) of individual fibers associated with onset of fiber atrophy. In comparison, mitochondrial peroxide generation was also increased in resting muscle from old (26 month) mice compared with adult (6–8 month) mice, but no age effect on fiber cytosolic H_2_O_2_ *in vivo* was seen. Thus ageing is associated with an increased ability of muscle fibers to maintain cytosolic redox homeostasis in the presence of denervation-induced increase in mitochondrial peroxide generation.

## Introduction

Age-related loss of muscle mass and strength is a disabling factor in older subjects for which approaches to prevention and therapeutic intervention are currently limited. In humans the inability to maintain mobility and physical function with age is thought to arise from a variety of molecular, cellular, endocrine and nutritional pathways, but studies also indicate that fundamental aging mechanisms play a key role in age-related loss of muscle mass and function. When loss of skeletal muscle becomes sufficient to affect the ability of individuals to limit normal function, it is known as sarcopenia. Estimates of the prevalence of this syndrome are variable, but may approach up to 13% in subjects over 60 years and as high as 50% those individuals over 80 years and is a major contributor to frailty in the elderly^1,2^. The specific causes of age-related loss of muscle fibers are unknown but loss is associated with remodelling and the loss of motor units^3^.

Considerable work on the causes of the loss of skeletal muscle mass and function with aging has focussed on the potential roles of motor neurons and disruption of neuromuscular junctions (NMJ) (see review by Hepple and Rice^4^). Loss of motor units occurs with aging in humans and animals^5,6^ and some studies indicate this loss of motor units occurs prior to loss of skeletal muscle mass^7^. Age-related loss of motor neurons with disruption of NMJ has been proposed to occur at least initially through changes in the peripheral motor axons since minimal loss of axons in the spinal cord has been reported in rodents which show disruption of NMJ^8–11^. There is evidence that throughout the life course, motor unit turnover occurs through cycles of denervation and re-innervation of individual muscle fibers and with advancing age these processes may eventually breakdown^12^. In young experimental animals the processes of motor unit turnover can be mimicked by induction of reversible damage to the motor nerves, such as through nerve crush injury. In previous studies, our group^13^ and others^14,15^ have reported that nerve transection in mice leads to increased generation of hydrogen peroxide (H_2_O_2_) and other peroxides by mitochondria in the denervated muscle. We speculated that denervation-induced increases in mitochondrial reactive oxygen species (ROS) production by individual muscle fibers might contribute to the increased ROS generation by muscle mitochondria that has been reported to occur during aging^13,16^, but whether such changes occur during motor unit remodelling is unknown.

Previous studies of mitochondrial H_2_O_2_ production from denervated muscle fibers have questioned the specificity of the amplex red technique to monitor H_2_O_2_ release in muscle since Bhattacharya, *et al*.^15^ and Pollock, *et al*.^13^ both showed that only a proportion of the amplex red oxidation could be inhibited by addition of catalase. Furthermore Bhattacharya, *et al*.^15^ attributed a substantial proportion of the amplex red oxidation to lipid peroxides released from mitochondria. Assessment of specific ROS, such as H_2_O_2_, in biological samples has been limited by the availability of suitable probes^17–19^, but in recent years a series of genetically encoded probes that are specific for H_2_O_2_ have been developed including HyPer^20^ and HyPer2, which has increased pH stability and a greater dynamic range for H_2_O_2_ detection^21^. These probes offer the potential to monitor H_2_O_2_ *in vivo* in accessible tissues and cells and have been used to demonstrate specific redox signalling and pathological roles of H_2_O_2_^22–26^. HyPer based probes have been sparsely used to study skeletal muscle in part due to the difficulties of transfection of this post-mitotic tissue. We have previously transfected isolated mature mouse skeletal muscle fibers with HyPer using a rAAV6 vector to monitor cytosolic H_2_O_2_ concentrations following contractile activity^24^ but analogous *in vivo* studies have not been reported.

The aims of the current studies were therefore to develop and optimise a technique to express the HyPer2 probe localised to the cytosol (cyto-HyPer2) in skeletal muscle of mice *in vivo* using an AAV vector, to allow serial monitoring of cytosolic H_2_O_2_ content in individual fibers of skeletal muscle *in vivo* using *intra vital* confocal microscopy. Following development of the method, this approach was used to examine the effect of nerve crush (as a model of motor unit turnover) on skeletal muscle cytosolic H_2_O_2_ content *in vivo* in comparison with *ex vivo* measurements of skeletal muscle mitochondrial peroxide generation obtained using the amplex red technique and to examine both mitochondrial and cytosolic H_2_O_2_ content in adult and old mice.

## Materials and Methods

### Experimental animals

Adult male Thy1-CFP (6–8 months old) mice were obtained from Jackson Laboratories (Maine, USA). These mice express cyan fluorescent protein (CFP) in motor and sensory neurons as well as a subset of central neurons to allow ready visualisation of nerves. Mice were housed in a temperature-controlled room (22–25 °C) with food and water *ad libitum* on a 12-h light/dark cycle in the University of Liverpool animal facilities. A sub-group of mice were also maintained in these facilities until 26 months of age (old mice). All experimental procedures were performed under a UK Home Office licence (Home Office licence number P391895CA, approved 15/06/17) and complied with the UK Animals (Scientific Procedures) Act 1986 and were ethically approved by the University’s Animal Welfare Committee (AWERB) (Ethical approval number AWC0066, approved 23/3/17).

### Transfection of muscles fibers with cyto-HyPer2

HyPer2 plasmid (Evrogen, Russia) was encased into an AAV6 viral vector (Vector Biolabs, USA) under a cytomegalovirus (CMV) promoter and inserted into the ECORV site in the multiple cloning site (MCS). In order to transfect the muscles for H_2_O_2_ detection, 50 µl of 5 × 10^10^ vg/ml (viral genome/ml of physiological saline) of rAAV6-HyPer2-cyto was injected directly into the anterior tibialis (AT) muscle of the mouse hind limb under gaseous anaesthetic (3% isoflurane knock down, then maintained at 1.75–2.5% isoflurane in 100% oxygen via nose cone until injection complete) at 28 days prior to study. This dosage regimen was based on the work of Chamberlain and colleagues^27^ and preliminary studies undertaken in our laboratory^24^.

### Procedure for peroneal nerve crush

Adult Thy1-CFP mice were anaesthetised using isoflurane, the hind limb was shaved, videne (antiseptic skin cleanser) applied and buprenorphine given (0.3 mg/ml) for anaesthesia. All animals were maintained under gaseous isoflurane anaesthesia throughout. A small incision was made on the outer side of the limb and the peroneal nerve exposed. The nerve was consistently crushed using a curved micro needle holder for 10 seconds, when a translucent band was visible across the nerve at the site of crush^28^. The surrounding connective tissue was then placed back over the nerve and skin sutured and the mice allowed to recover in a heated chamber until normal movement, feeding and exploratory behaviour were observed. Groups of mice were allowed to recover for 3, 7 or 21 days before being sacrificed when tissues were dissected and used for histological analyses. A further group were used for *intra vital* imaging and were serially studied at 3, 7 and 21 days following nerve crush and were sacrificed following study at 21 days when tissues were dissected. Sham surgery was also undertaken to provide appropriate controls for both groups and this included exposure of the peroneal nerve without nerve crush.

### *Intra vital* imaging

**Figure 1:**
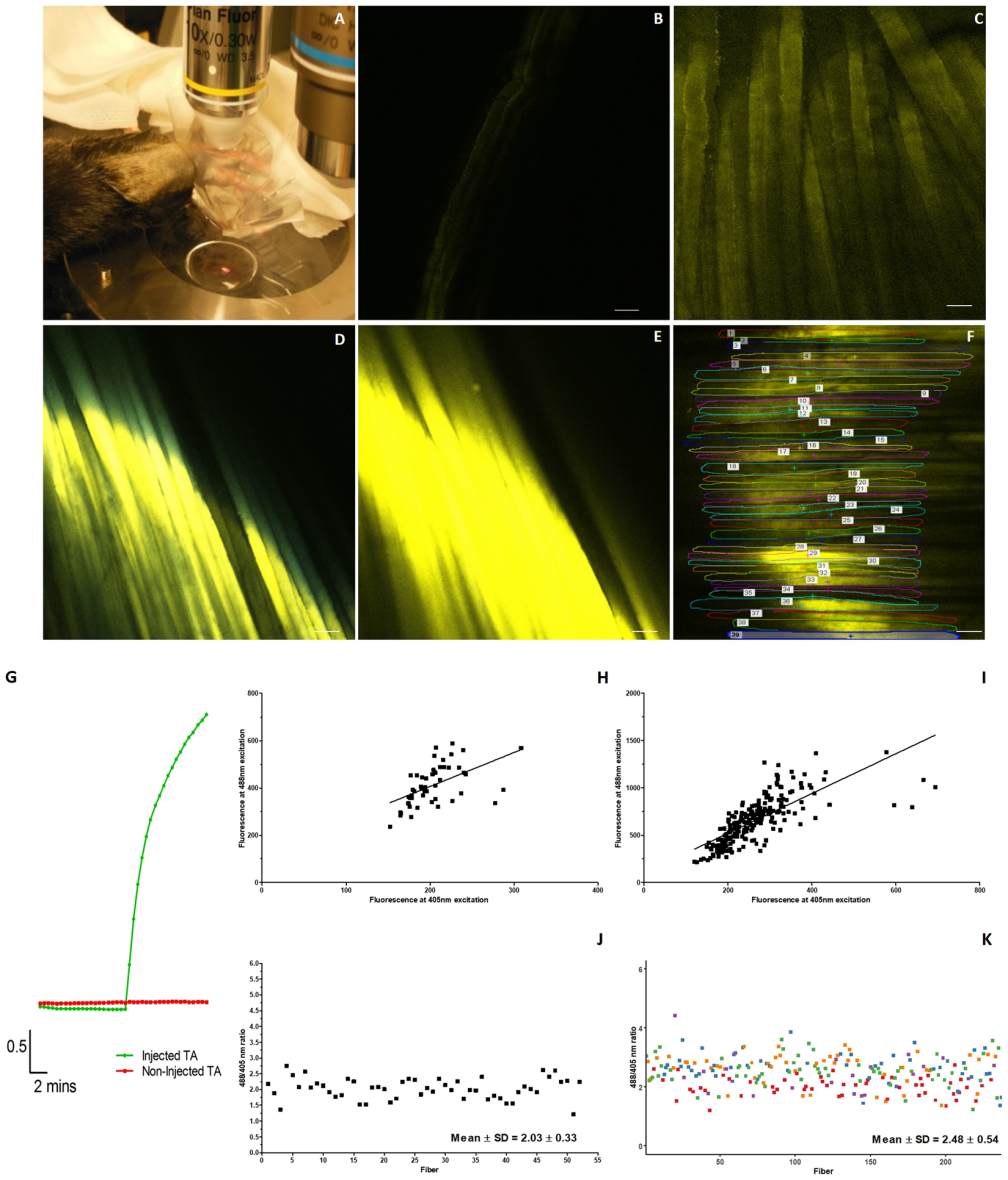
Intra vital microscopy set up for HyPer2 visualisation. (**A**) Typical intra vital microscope set up for imaging AT WT muscle *in vivo*. Representative image of AT muscle at 28 days following transfection with rAAV6 vector (**B**) without cyto-HyPer2 insert, and (**C**) with the cyto-HyPer2 plasmid inserted using rAAV6 vector confirming wide spread fluorescence from HyPer2 transfection. Typical fluorescence image of HyPer2 transfected *ex vivo* muscle preparation (**D**) prior to addition of exogenous H_2_O_2_ and (**E**) 5 mins post addition of exogenous H_2_O_2_. (**F**) Image analysis outlining the identification of regions of interest (ROI) in individual fibers from one AT muscle for HyPer2 fluorescence. Graphical representations of HyPer2 ratiometric analysis showing (**G**) time course of change in the ratios of emissions at 516 nm following excitation at 488 nm and 405 nm (488/405 ratio) for H_2_O_2_ treated and non-treated muscle *ex vivo*. Comparison of ratio emissions at 516 nm after 488 and 405 excitation for (**H**) 52 fibers in a single AT muscle (Pearson r = 0.53, p < 0.001) and (**I)** from 237 fibers across 5 AT muscles from different mice (Pearson r = 0.74, p < 0.001). Graphical representation of the ratio obtained from the (**J**) ROI of 52 fibers in a single AT muscle and (**K**) from the ROI of 237 fibers from 5 AT muscles from different mice – different colours correspond to the AT muscles of 5 different mice, scale bar for all images = 100 µm).

Under isoflurane anaesthesia, mice were placed on their side before a custom made translucent well-like structure was placed on the limb (Fig. 1A). The limb was then bathed in a physiological saline that contained (mM): NaCl 150, KCl 5, CaCl 2, MgCl_2,_1, TES buffer 2 (pH 7.4)^29^. A Nikon x10 water immersion lens was lowered into position and focussed on the surface of the AT muscle. With the well in place, an incision was made to expose the AT muscle, the epimysium was removed to allow visualisation of multiple superficial individual skeletal muscle fibers, their NMJ’s and the peripheral motor neuron innervating the fiber. Following capture of images, the well was removed, the skin was sutured and mice were allowed to recover. The process was repeated with imaging of the same area of the AT at 3, 7 and 21 days post-crush. All serial imaging *in vivo*, in addition to imaging of fixed samples, was performed using a Nikon A1R confocal microscope utilising the acquisition software Nikon NIS Elements AR V.4.40 (64 bit). CFP was excited at 405 nm with emission collected at 485 nm. HyPer2 was sequentially excited at both 405 and 488 nm with emissions collected on the 516 nm PMT detector; the ratio of emissions at 516 nm after 488 and 405 excitation are presented as a measure of H_2_O_2_ content that is independent of HyPer2 content. This dual approach using Thy-1 CFP and HyPer2 fluorescence was followed to enable estimation of the H_2_O_2_ content together with visualisation of the structure of the pre-synaptic NMJ to be undertaken serially. For *intra vital* studies all micrographs were captured with the x10 objective using the Galvano scanner at 512 × 512 dimensions with a 33.2 μm pinhole. Where samples were examined *ex vivo*, fixed sample micrographs were captured by z stack using the x60 or x10 objectives using the Galvano scanner at 512 × 152 dimensions with a 43.42 μm pinhole and taken at 5 μm steps through the tissue and captured with a scanner zoom of 2.00. All experiments were performed at 25°C.

### Assessment of skeletal muscle fiber morphological changes

The anterior tibialis (AT) was excised and embedded in OCT (Thermo-Scientific, Cheshire, UK) and frozen in isopentane cooled by liquid nitrogen. Transverse sections were cryosectioned at a thickness of 12 µm (Leica CM1850, Germany) and transferred to superfrost plus slides (Thermofisher, UK). Sections were stained using Hematoxylin and eosin. Sections were mounted in vectashield hardset mountant with DAPI (Vector labs, Peterborough, UK). Muscle sections were imaged on a Nikon Eclipse TE2000-5 inverted microscope using the Hamamatsu C-4742-95 camera with IPLab software. Images were analysed to find the minimal Feret’s  diameter of 2220 ± 579 fibers (mean + -SD) per AT using ImageJ (NIH), (*n* = 4 animals per treatment group).

### *Ex vivo* assessment of NMJ morphology and analysis of fragmentation

Following dissection, the AT muscle was fixed at approximate *in situ* length, pinned and fixed for 2hrs with 10% neutral buffered formalin (Leica, UK) before being stored in PBS-Sodium Azide. All samples were stained using α-bungarotoxin Alexa Fluor-594 (10 μg/ml, Invitrogen) for 1 hr at room temperature to visualise acetylcholine receptors (AChR). After washing with PBS, the AT muscle was then pinned and imaged using the Nikon A1R intra vital microscope using multi-dimension acquisition software to capture 5 µm z-stacks through several regions of the muscle to obtain representative images of the innervation status of the AT muscle. All micrographs were analysed using the NIS elements software using regions of interest and digital zoom for ultrastructure visualisation of individual NMJs. NMJ fragmentation in adult mice post-crush was classified according to the number of postsynaptic fragments that were observed at each NMJ: Score 1 = normal NMJ without fragmentation; Scores 2–4 = light fragmentation of the pre-synaptic terminal retaining a pretzel-like post-synapse; Scores > 5 = intermediate to severe fragmentation^30^.

### Analysis of HyPer2 fluorescence from individual muscle fibers

All analysis was masked to surgical treatment and analysis techniques were based upon approaches previously described (Pearson *et al*., 2014) whereby individual regions of interest (ROIs) were selected and the HyPer2 fluorescence calculated (see Fig. 1F for example). All fibers were individually circled as ROIs using the Nikon NIS Elements 4.40 64 bit software.

### *Ex vivo* measurements of mitochondrial peroxide production from bundles of permeabilised AT fibers

Small bundles of muscle fibers from the AT muscle were permeabilised and examined to assess the rate of mitochondrial generation of H_2_O_2_ and other peroxides^13^. Following rapid dissection, the AT muscle was cut into smaller bundles using a scalpel and then placed immediately into 200 μM Saponin (Sigma Aldrich, UK) in relax buffer (containing (mM): MgATP 4.5, Free Mg 1, Imidazole 10, EGTA 2 and KCl 100, pH 7.0) for 8 mins at RT to permeabilise the fibers as described by Pollock, *et al*.^13^. All fibers were then washed several times with relax buffer before being placed into Amplex Red solution (37.5U/mg SOD, 19.44 mM Amplex red, 5U/ml Horse radish Peroxidase (HRP) in ROS buffer) in black 96 well plates (Corning, Wiesbaden, Germany). Formation of the fluorescent product resofurin red was followed at 590 nm using a fluorimeter (Fluorstar, BMG Labteck, Germany) at 37 °C^31^. All values were then normalised for the protein content of the fiber bundles which was obtained using the Bradford assay. The rate of peroxide production was expressed as pmoles H_2_O_2_/min/mg protein.

### Statistical analysis

Data are presented as mean ± SEM unless stated otherwise, and *P* < 0.05 was considered significant with asterisks to denote level of significance as *p < 0.05, **p < 0.01 and ***p < 0.001. All ROI statistics for all wavelengths were exported and further analysed using GraphPad Prism 5.0 (GraphPad, CA, USA). The HyPer ratio of emissions obtained with 488 nm excitation/405 nm excitation was calculated and all data are expressed as mean ± S.E.M for each ROI unless stated otherwise. For HyPer optimisation studies (Fig. 1H,I), Pearson correlations were calculated and two tailed statistical analysis was performed and all R values and P values reported. For all muscle weight and body mass analyses One-way ANOVAs with Bonferroni post-hoc tests we performed. For NMJ fragmentation analysis, a Two-way ANOVA with Bonferroni post hoc test was carried out. Intra vital imaging post crush and mitochondrial peroxide content used One-way ANOVA with Tukey’s multiple comparison tests.

## Results

### Transfection of skeletal muscle with HyPer2 and development of an intra vital assay to assess H_2_O_2_ in skeletal muscle fibers

#### Efficacy of the AAV6 approach to transfect muscle with HyPer2 *in vivo*

Following previous studies undertaken to transfect skeletal muscle fibers with HyPer using rAAV6 *ex vivo*^24^ and *in vivo* studies undertaken by Chamberlain and colleagues^27^, we examined the efficacy of the AAV6 approach to transfect AT fibers with HyPer2. *Ex vivo* images of the AT muscle from mice at 28 days post-injection indicate that the vast majority of the AT fibers expressed fluorescent HyPer2 in comparison with the empty vector-injected control mice where very little fluorescence was observed (Fig. 1B,C).

#### Response of the transfected AT muscles to H_2_O_2_

Following sacrifice, the AT muscle of the hind limb of a transfected mouse was exposed and bathed in physiological saline solution (Fig. 1A) and imaged every 30 seconds for 20 mins. After 10 minutes of recording, H_2_O_2_ was added to the bath to achieve a final extracellular concentration of 500 μM. Figure 1D,E,G show a rapid and significant increase in the fluorescence from H_2_O_2_-treated muscle and ratio of emissions at 516 nm after 488 and 405 excitation following H_2_O_2_ challenge in those mice where the AT muscle had been injected with rAAV6-Hyper2 at 28 days previously with no change in the control non-transfected mice.

#### Optimisation of intra vital microscopy

Following demonstration of the efficacy of transfection and response to exogenous H_2_O_2_, we studied HyPer2 fluorescence from the AT muscle *in vivo* using *intra vital* microscopy. Initial images were very poor with very weak fluorescence detected (see Supplementary Data, Fig. S1(A,B)) such that neither CFP fluorescence from the Thy1-CFP labelled neurons nor YFP fluorescence from the HyPer2 could be detected. Removal of the epimysium (the collagen sheath that covers the muscle) produced a substantial improvement in the quality of the intra vital images and level of fluorescence detected (Fig. S1(C,D)) and the epimysium was routinely removed for all further studies.

Obtaining high quality *intra vital* images from the AT muscle was compromised by movement in the live mouse (a typical example is shown in Supplementary Video, Fig. S2). This movement occurred despite precautions taken to stabilise the muscle without compromising tissue blood flow. Images were therefore taken as rapidly as possible to minimise movement artefact. The main purpose of the *intra vital* protocol was to analyse the HyPer2 signal, images were taken so that the muscle fibers were in focus. As such NMJs were normally out of focus during this stage of our protocol, however, owing to the unique structure of the NMJs and neuronal innervation *ex vivo* images from the same regions could be captured and NMJs evaluated in detail.

#### Reproducibility of HyPer2 fluorescence from individual muscle fibers

In order to examine the variability in cyto-HyPer2 fluorescence (and hence cytosolic H_2_O_2_ content) between fibers of the same AT muscle *in vivo* where only rapid image acquisition was possible, we examined the relationship between emissions with excitation 488 nm and those obtained with 405 nm excitation from 51 surface fibers that were readily visible in one mouse AT muscle (Fig. 1H). These data indicate that emissions obtained at 405 nm excitation varied by up to 2 fold (potentially indicating substantially different levels of expression of the probe), but the ratio of emissions with 488 and 405 nm excitations (488/405 emission ratio) remained remarkably constant (Fig. 1J) across the different fibers with a mean ratio of 2.03 with a standard deviation of 0.33 indicating little variability in the ratio (and hence H_2_O_2_ content) between fibers of the same AT muscle *in vivo*.

It was also important to examine the differences that were present between muscles from different mice since these will be potentially influenced by factors such as quality of the measurements undertaken and individual biological variability. This was again assessed by comparing the emission obtained following 488 nm excitation and that from 405 nm excitation from fibers of 5 AT muscles from different adult mice (Fig. 1I) (237 fibers in total). The HyPer2 probe is ratiometric and hence within a certain range the ratio of emissions at 516 nm after 488 and 405 excitation should not be influenced by probe concentration. We observed a good correlation between the emission values at the 2 excitation wavelengths (Fig. 1I, Pearson r = 0.53, p < 0.001) even though the emissions at 405 nm excitation varied between fibers (i.e. potentially reflecting variability in probe expression between fibers). These data also indicate a relatively low variability in cytosolic H_2_O_2_ content between fibers and between muscles of adult mice indicated by the relatively consistent ratio of emissions at 516 nm after 488 and 405 excitation (Fig. 1K, Pearson r = 0.74, p < 0.001).

#### Effect of peroneal nerve crush on NMJ structure and skeletal muscle atrophy

**Figure 2:**
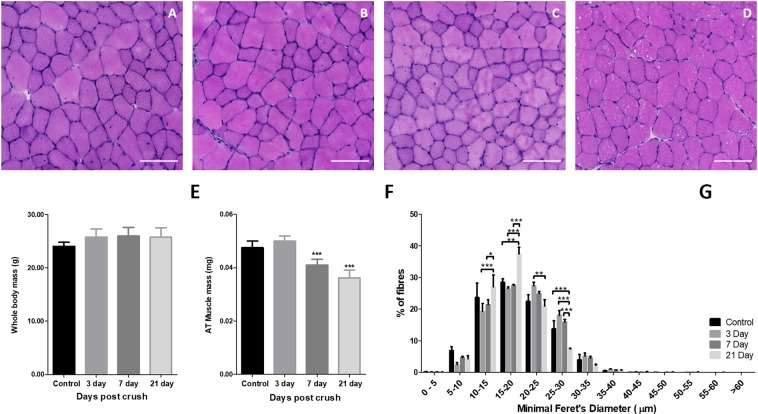
Muscle morphological analysis post nerve crush. Muscle fiber morphology from the AT muscle prior to, and at 3, 7 and 21 days post nerve crush post crush visualised from (**A**–**D**,**H**,**E**) stained transverse sections (scale bar = 100 µm). (**E**) Mouse body weights revealed no significant differences over the time course (p = 0.10, One way ANOVA, *n* = 6), (**F**) however AT muscle mass was significantly decreased from 7 days post crush (p < 0.001, One way ANOVA with Bonferroni post hoc test, *n* = 6), (**G**) fiber size distribution where assessed using Minimal Feret’s diameter with significant differences shown (p < 0.05, Two way ANOVA, *n* = 4).

**Figure 3:**
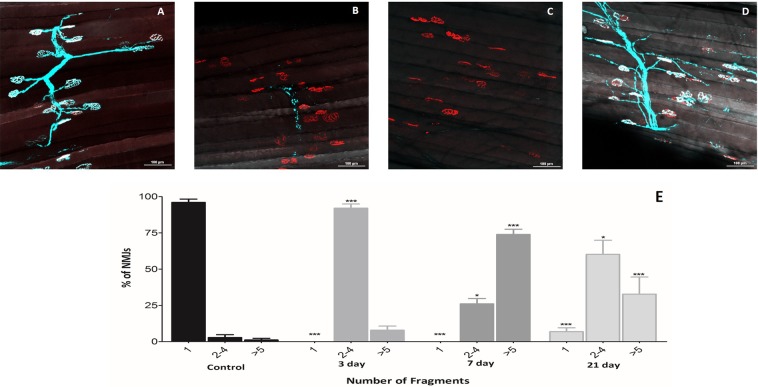
Neuromuscular junction morphological analysis post-nerve crush. N.M.J. morphology from the A.T. muscle *ex vivo* prior to, and at 3, 7 and 21 days post nerve crush (**A**–**D** respectively) from Thy1-CFP mice to visualise pre-synaptic terminal motor nerves (blue) and counterstained with α-bungarotoxin staining to reveal post synaptic AChR clusters (red). (**E**) NMJs were classified prior to, and at 3, 7 and 21 days post nerve crush into three categories, according to the number of postsynaptic fragments observed (**A**–**E**) 1 = normal NMJ without fragmentation; 2–4 fragments = light fragmentation with pretzel-like postsynapse; >5 fragments = intermediate to severe fragmentation. The data revealed significant fragmentation beginning at 3 days post nerve crush with some recovery at the later time point (*p < 0.05, ***p < 0.001, Two-way ANOVA with Bonferroni post hoc test, *n* = 4 animals,approximately 250–350 endplates were assessed in total at each time point. Scale bar = 100 µm for all images.

Nerve crush was used as an experimental model to induce motor unit turnover. The data shown in Figs 2 and 3 illustrate the temporal effect of crush on the nerve and muscle structure over 21 days post-crush. Representative transverse sections of the AT muscle prior to, and at 3, 7 and 21 days post-crush are shown in Fig. 2A–D (H&E stain). Mouse body weights and AT muscle mass are shown in Fig. 2E,F and the muscle fiber minimal Feret’s diameter is shown in Fig. 2G. These data illustrate an effect of nerve crush on muscle atrophy that was apparent by 7 days post-crush and remained significant at 21 days with no significant effect on mouse body weight. The data in Fig. 2G show that this was associated with a tendency to a reduced number of larger fibers at 21 days post-crush. Figure 3A–D are representative images illustrating the effect of the nerve crush on the pre-and post-synaptic structure of the NMJ prior to, and at 3, 7 and 21 days post-crush. It is apparent that the crush caused fragmentation and loss of the pre-synaptic structure by 3–7 days post-surgery and that this had partially reversed by 21 days. Quantification of the degree of pre-synaptic fragmentation is shown in Fig. 3E which shows some reversal of the fragmentation by 21 days although this was incomplete.

#### Intra vital assessment of the effect of nerve crush on cyto-HyPer2 fluorescence from AT muscle fibers

**Figure 4:**
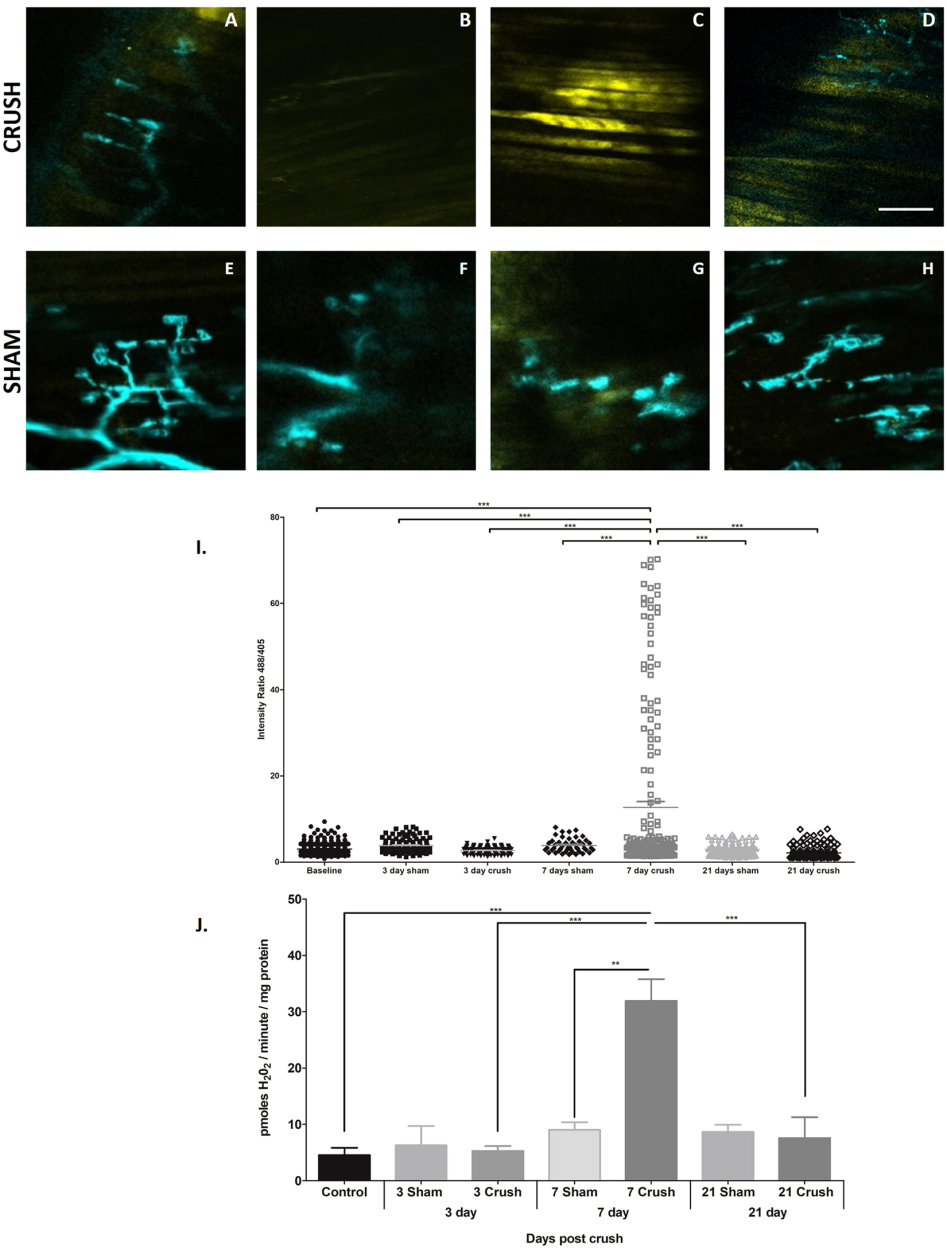
*Intra vital* HyPer2 analysis post-nerve crush in adult mice and mitochondrial peroxide generation from permeabilised fibers. Representative serial intra vital images of the same area of an AT muscle transfected with rAAV6-cyto-HyPer2 from a Thy1-CFP mouse *in vivo* demonstrating (**A–D**) HyPer2 fluorescence prior to, and at 3, 7 and 21 days post-nerve crush with (**E–H**) the effects of sham surgery whereby the nerve was kept intact and the Thy1-CFP signal remained unaltered (scale bar = 100 µm for all images). (**I**) HyPer2 ratio of emissions at 516 nm following excitation at 488 and 405 nm (488/405 ratio) prior to, and at 3, 7 and 21 days post-nerve crush revealed a significant increase at 7 days post crush compared to all other times points and sham treatments (***p < 0.001, One-way ANOVA with Tukey’s multiple comparison test, *n* = 10 for crush and sham) Data for individual fibers in (I) revealed that approximately 24% of fibers at 7 days post crush were responsible for the elevated average 488/410 ratio. In line with previous published data, mitochondrial peroxide production was assessed by (**J**) rate of amplex red oxidation during state 1 respiration (i.e. in the absence of added substrates) from permeabilised AT fibers prior to, and at 3, 7 and 21 days post-nerve crush revealed significant increases at 7 days post crush (**p < 0.01,***p < 0.001, One-way ANOVA with Tukey’s multiple comparison test, *n* = 4 for each group).

Figure 4A–D are representative serial images of the same area of fibers from the AT muscle from one mouse *in vivo* prior to, and at 3, 7 and 21 days post-nerve crush with images of the sham-operated control AT muscles in Fig. 4E–H. Mean changes in the ratio of emissions at 516 nm after 488 and 405 excitation at these time points are shown in Fig. 4I. The images of CFP fluorescence from pre-synaptic structures shown in Fig. 4A–D are lacking in fine detail (as discussed previously) but fragmentation and loss of pre-synaptic structures are apparent at 3 and 7 days in accord with the *ex vivo* images in Fig. 3. The mean data from HyPer2 fluorescence shows a substantial and significant increase in the ratio of emissions at 516 nm after 488 and 405 excitation at 7 days post-crush (Fig. 4I) that was not apparent at other time points or in the sham operated mice. The ratio of emissions at 516 nm after 488 and 405 excitation from individual fibers are also shown in Fig. 4I. This reflects the large increase in mean values shown in Fig. 3I, but surprisingly the large increase seen in the muscles at 7 days post-nerve crush appears to be confined to ~24% of the total fibers that were assessed. Peroxide release from mitochondria in state 1 are presented in Fig. 4J and also show a significant increase at 7 days post-nerve crush. These data show peroxide generation in state 1 respiration and data for mitochondria treated with various substrates and inhibitors of the electron transport chain (ETC) are presented in Supplementary Fig. S3.

#### Intra vital assessment of cyto-HyPer2 fluorescence from the AT muscle of adult and old mice

**Figure 5:**
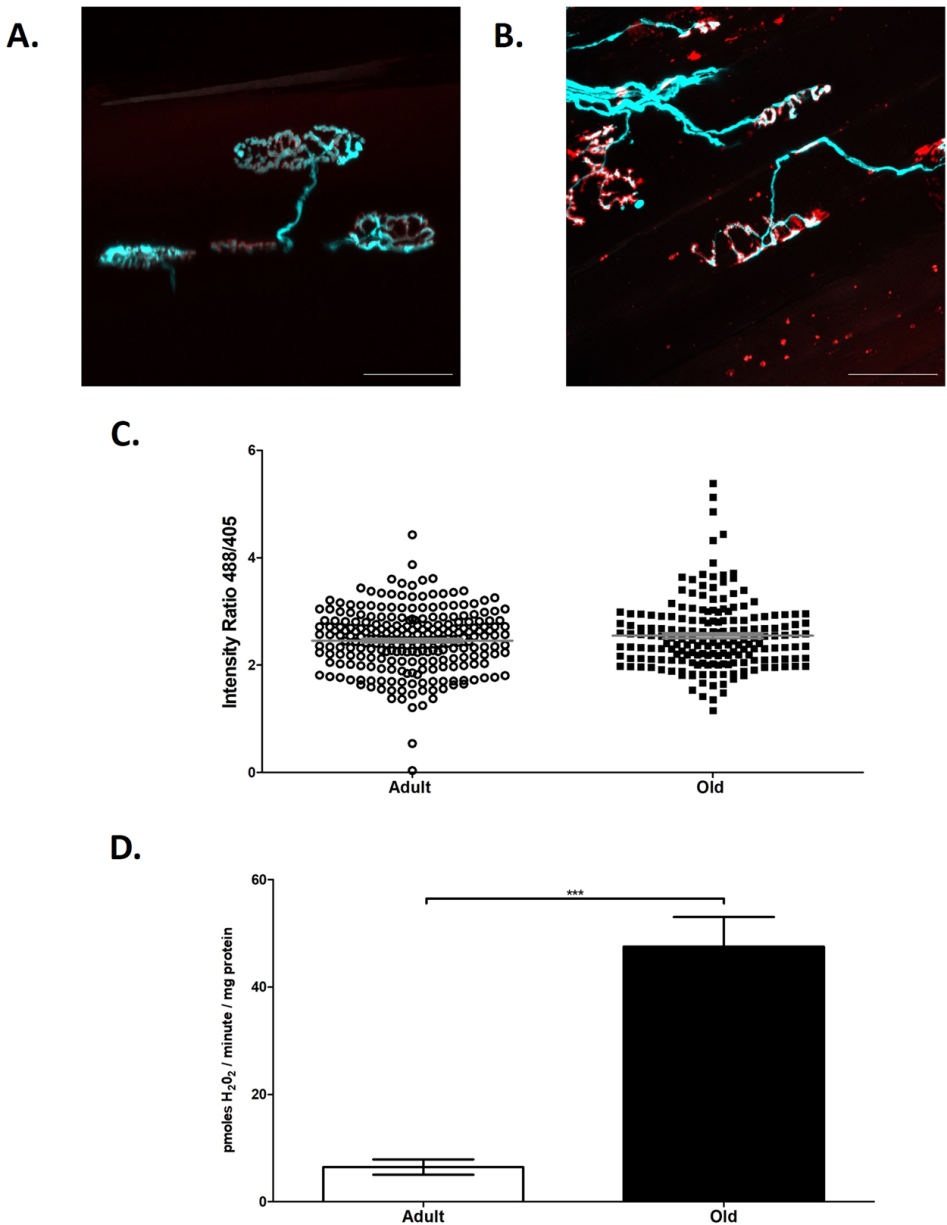
Neuromuscular junction, HyPer2 *intra vital* assessment and mitochondrial peroxide generation; comparison between adult and aged mice. Representative fluorescent images of NMJ’s from adult (6–8 months) (**A**) and old (26 months) (**B**) Thy1-CFP mice showing the pre-synaptic terminal motor nerves (blue) and motor endplates stained with α-bungarotoxin (red) (scale bar = 50 µm). No significant difference was observed between HyPer2 transfected AT muscles from adult and old mice when assessing the ratio of emissions at 516 nm after 488 and 405 excitation (488/405 ratio) (**C**) from individual fiber ratios. (**D**) Rate of amplex red oxidation (expressed as H_2_O_2_ generation) during state 1 respiration (i.e. in the absence of added substrates) from permeabilised AT fibers from adult and old mice (***p < 0.001, Mann Whitney, n = 11 for adult, n = 7 for old).

Figure 5 shows representative *ex vivo* images of Thy-1 CFP and bungarotoxin labelled NMJs from adult (A) and old (B) mice. Mean values for the 488/405 ratio from cyto-HyPer2 fluorescence *in vivo* are shown in Fig. 5C with no significant differences between the groups. The data from individual fibers are presented in Fig. 5C and these generally reflect the lack of difference between groups although a small number of fibers from the AT muscle of old mice showed individual values higher than any fibers in the adult mice. In contrast, Fig. 5D shows *ex vivo* measurements of state 1 peroxide release from bundles of permeabilised fibers from adult and old mice and these show a significant increase in peroxide production from mitochondria obtained from muscles of old (26 months) compared with adult (6–8 months) mice.

## Discussion

This study has used a novel approach to monitor cytosolic H_2_O_2_ content in individual fibers in the AT muscle of mice *in vivo*. The approach developed using HyPer2 has demonstrated that a proportion of AT fibers show an increase in cytosolic H_2_O_2_ concentration following peroneal nerve crush in a situation where mitochondria appear to be releasing substantially increased amounts of peroxides. This lack of any direct correlation between *ex vivo* mitochondrial peroxide release and *in vivo* cytosolic H_2_O_2_ content was even more marked in old mice which show increased mitochondrial peroxide release in comparison with adult mice, but little evidence of increased cytosolic H_2_O_2_ content. Thus the data presented demonstrate the importance of studying specific reactive species in single fibers *in vivo*, since the pattern of changes in cytosolic H_2_O_2_ content observed could not be implied from *ex vivo* data obtained from studies of muscle mitochondria.

These studies have utilised a rAAV6 vector to transfect the HyPer2 probe into skeletal muscle *in vivo*. The use of AAV’s has previously proved effective in transfection of mouse muscle *in vivo* in studies aimed at replacement of dystrophin in mdx mouse muscles^32–34^ and has also been used to transfect mouse muscle fibers *ex vivo* with HyPer probe^24^. Images indicate that >90% of AT muscle fibers show YFP fluorescence following the AT single intramuscular injection protocol (Fig. 1) and measurements of the 488/405 ratio show the reproducibility of the fluorescence measurements from the visible surface fibers of a single transfected muscle (Fig. 1K). The data in Fig. 1 also shows the rapid response of the transfected HyPer2 to exogenous addition of H_2_O_2_ to the AT *ex vivo* (Fig. 1D,E,G). The time course of the response indicates a substantial increase in fluorescence within 1 minute of addition of H_2_O_2._ This time course must reflect the time taken for diffusion of the H_2_O_2_ into the muscle fiber in addition to the response time required for probe oxidation.

Reproducibility of the HyPer2 measurements between different muscle fibers of the same muscle was assessed by examining the 488/405 ratio in multiple fibers and showed remarkably consistent results (Fig. 1H,J). In comparisons between 237 fibers from muscles of 5 different mice, the emission following 405 nm excitation varied approximately 10 fold which may reflect differing levels of expression of the HyPer2 probe, but the relationship between the emission at 488 nm and that at 405 nm remained consistent, providing reassurance of the robustness of the ratiometric probe and also indicates a lack of major variations in cytosolic H_2_O_2_ content between muscle fibers from control adult mice at rest.

The HyPer2 probe was used to monitor the effect of peroneal nerve crush on muscle cytosolic H_2_O_2_ content. The crush protocol used was found to induce some loss of pre-synaptic NMJ structure by 3 days and by 7 days post-crush all of the peripheral motor neurons and pre-synaptic NMJ had been lost. By 21 days there appeared to be some recovery of the pre-synaptic structures although this was incomplete with significant fragmentation still present (Fig. 3E). Despite the loss of pre-synaptic structure, the post-synaptic AChR remained intact throughout the 21 day study retaining the typical “pretzel-like” structure seen prior to nerve crush. The nerve crush also induced a loss of muscle mass within 7 days which was a similar time course to that seen following peroneal nerve transection (Pollock *et al*.^13^) and the muscle loss was maintained up to 21 days despite the apparent partial restoration of motor nerve and NMJ structures at that time point.

The procedure for repeated imaging of the same area of the muscle at 3, 7 and 21 days later proved entirely feasible as reported by others^35^, but in some animals from both the experimental and control groups the repeat surgery and suturing was found to induce scar tissue formation which led to some increase in autofluorescence (commonly produced by lipofusins, NADPH and flavins^36,37^). This increase in scarring and possible autofluorescence did not appear to significantly affect the ratio of emissions at 516 nm after 488 and 405 excitation since values from sham-operated mice showed no significant change with time (Fig. 4). In contrast the mean 488/405 ratio from muscles of mice which had undergone the nerve crush showed a large and significant increase at 7 days post crush that had returned to baseline values by 21 days. Thus the apparent increase in cytosolic H_2_O_2_ content occurred in association with atrophy of the muscle fibers, but had reversed by 21 days although at this stage the loss of fiber area remained significant and there was still evidence for loss of NMJ integrity. The mean 488/405 ratio data do not fully reflect the pattern seen in individual muscle fibers (Fig. 4I) which shows a clear substantial increase in HyPer2 fluorescence in only ~24% of the fibers at 7 days post-crush, although all fibers had undergone nerve crush.

Mean data for mitochondrial peroxide release also showed a significant increase at 7 days post-crush (Fig. 4J). The mitochondrial electron transport chain is known to be a major source of cellular oxidative stress^15,38,39^ and studies from our group and others have reported that ROS production was elevated from mitochondria in atrophied and denervated skeletal muscles^13,14,40–42^. Many of these studies have used the amplex red technique to examine H_2_O_2_ generation from isolated mitochondria and have assumed that oxidation of amplex red specifically reflects H_2_O_2_ release from mitochondria. Recent papers have suggested that in denervated muscle mitochondria, release of lipid peroxides may also play a role in the amplex red oxidation^13,15^.

The lack of a direct correlation between data on cytosolic H_2_O_2_ content and mitochondrial peroxide release is undoubtedly due in part to the localisation of the HyPer2 to the fiber cytosol whereas in permeabilised fibers the amplex red oxidation reflects mitochondrial production of ROS. Thus the release from mitochondria may only have become sufficient to affect cytosolic H_2_O_2_ concentrations in some fibers at 7 days post-nerve crush. We have previously studied the extent to which release of ROS from mitochondria can influence cytosolic ROS in skeletal muscle and suggested that this is limited in intact muscle fibers^43^, however the extent to which increases in muscle mitochondrial H_2_O_2_ generation are reflected in cytosolic H_2_O_2_ concentrations in individual fibers does not appear to have ever been directly studied. The heterogeneity of response between individual fibers is surprising, but may be related to differences in levels of cytosolic regulatory proteins, such as the peroxiredoxins, or peroxidases that degrade H_2_O_2_. It is also feasible that the generation of peroxides by mitochondria and generation of H_2_O_2_ in the cytosol are both stimulated by denervation, but occur by different processes.

Wide spread application of the original HyPer probe for assessment of H_2_O_2_ was limited by a low dynamic range of responses and by a significant effect of pH on the fluorescence intensity. The HyPer2 probe used in the present study was developed to provide increased dynamic range and pH stability. The sensitivity of the isolated HyPer2 probe to H_2_O_2_ has been reported to be 20–30 nM^44^ which may be insufficiently sensitive to reliably detect physiological H_2_O_2_ responses in skeletal muscle^45–47^, but showed clear responses to higher exogenous increases in H_2_O_2_. Previous data using 31-P MR approaches indicate there are no significant changes in intracellular pH that occur in resting muscle following denervation^48^ and hence differences in pH between fibers are unlikely to have contributed to the pattern of responses in HyPer2 fluorescence seen.

The lack of clear correlation between the data from mitochondrial production of peroxides and cyto-HyPer2 was even more pronounced when old mice were studied (Fig. 4). Mitochondria from old mice showed increased generation of peroxides, as previously described^13,16,49^ although this had no significant effect on cyto-HyPer2 fluorescence. The extent of the overall increase in mitochondrial peroxide generation in old mice was roughly equivalent to that seen 7 days post nerve crush (Fig. 4K). It is relevant that muscle from old mice has been reported to display numerous increases in the content of cytosolic proteins that degrade peroxides, such as catalase and glutathione peroxidase 1^50^, peroxiredoxins^51^ and thioredoxins^52^. These increases appear to reflect an attempt by the aging tissue to adapt to protect against deleterious effects of increased oxidative stress and it seems likely that this provides an effective approach to maintain cytosolic redox homeostasis in the presence of elevated mitochondrial peroxide generation. The level of the mitochondrial peroxide release reported here for muscles of old mice was lower than that previously reported^13^. This is likely to reflect slight differences in age of the cohorts of mice studied and/or the level of denervation and disruption of NMJ in the specific ageing cohort^13^.

It is relevant to consider potential functions of the H_2_O_2_ generated in response to nerve crush. Most data have assumed that increased H_2_O_2_ is associated with, and mediates degenerative changes in tissues and Bhattacharya and colleagues (2014) previously demonstrated that inhibition of lipoxygenase-mediated enzymatic lipid peroxidation reduced muscle atrophy following denervation which supports this possibility. In contrast, a number of recent studies have demonstrated that increased H_2_O_2_ can facilitate repair of muscle^53^ and may be a stimulus for axonal growth and re-innervation since low levels of H_2_O_2_ have been reported to stimulate axonal sprouting in both mammalian and non-mammalian systems^54,55^.

In summary, this study has shown the feasibility of direct *in vivo* measurements of cytosolic H_2_O_2_ in muscle following nerve crush injury and has demonstrated that an overall increased mitochondrial peroxide generation and cytosolic H_2_O_2_ content is associated with muscle fiber atrophy. Detailed comparison of the changes in H_2_O_2_-specific HyPer2 oxidation in individual muscle fibers with amplex red oxidation from mitochondria of permeabilised muscle fibers show that only a sub-group of fibers respond to increased mitochondrial peroxide generation by increasing cytosolic H_2_O_2_ content. It is argued that the most likely explanation for this discrepancy is differing activities of proteins involved in H_2_O_2_ degradation between fibers. This differs from the situation in muscles of old mice where increased mitochondrial peroxide generation was not reflected in an increase in cytosolic H_2_O_2_ potentially reflecting an increased ability to degrade cytosolic peroxides that has occurred as muscle adapts to the increased oxidative stress of aging.

## Supplementary information

Supplementary information
