# Supplementary material for: HyPer2 imaging reveals temporal and heterogeneous hydrogen peroxide changes in denervated and aged skeletal muscle fibers in vivo
Source: Sci Rep. 2019 Oct 8;9:14461. doi: 10.1038/s41598-019-51035-w (PMC6783413; doi:10.1038/s41598-019-51035-w)
Supplement: Supplementary file 1 — Supplementary information [file 41598_2019_51035_MOESM1_ESM.pdf]

HyPer2 imaging reveals temporal and heterogeneous hydrogen peroxide changes in denervated and aged skeletal muscle fibers *in vivo.*

CA Staunton, ED Owen, N. Pollock, A Vasilaki, R Barrett-Jolley, A McArdle and MJ Jackson*

MRC-Arthritis Research UK Centre for Integrated research into Musculoskeletal Ageing (CIMA), Department of Musculoskeletal Biology
Institute of Ageing and Chronic Disease,
University of Liverpool,
Liverpool, L7 8TX,
U.K.

Supplementary information

Figure S1 Example CFP (**A**) and YFP (**B**) intra vital fluorescence images from the surface fibers of the AT muscle of HyPer2 transfected Thy1-YFP mice in the presence of the epimysium**.** Example CFP (**C**) and YFP (**D**) intra vital fluorescence images from the surface fibers of the AT muscle of HyPer2 transfected Thy1-YFP mice with the epimysium removed (scale bar for all images = 100µm)**.**

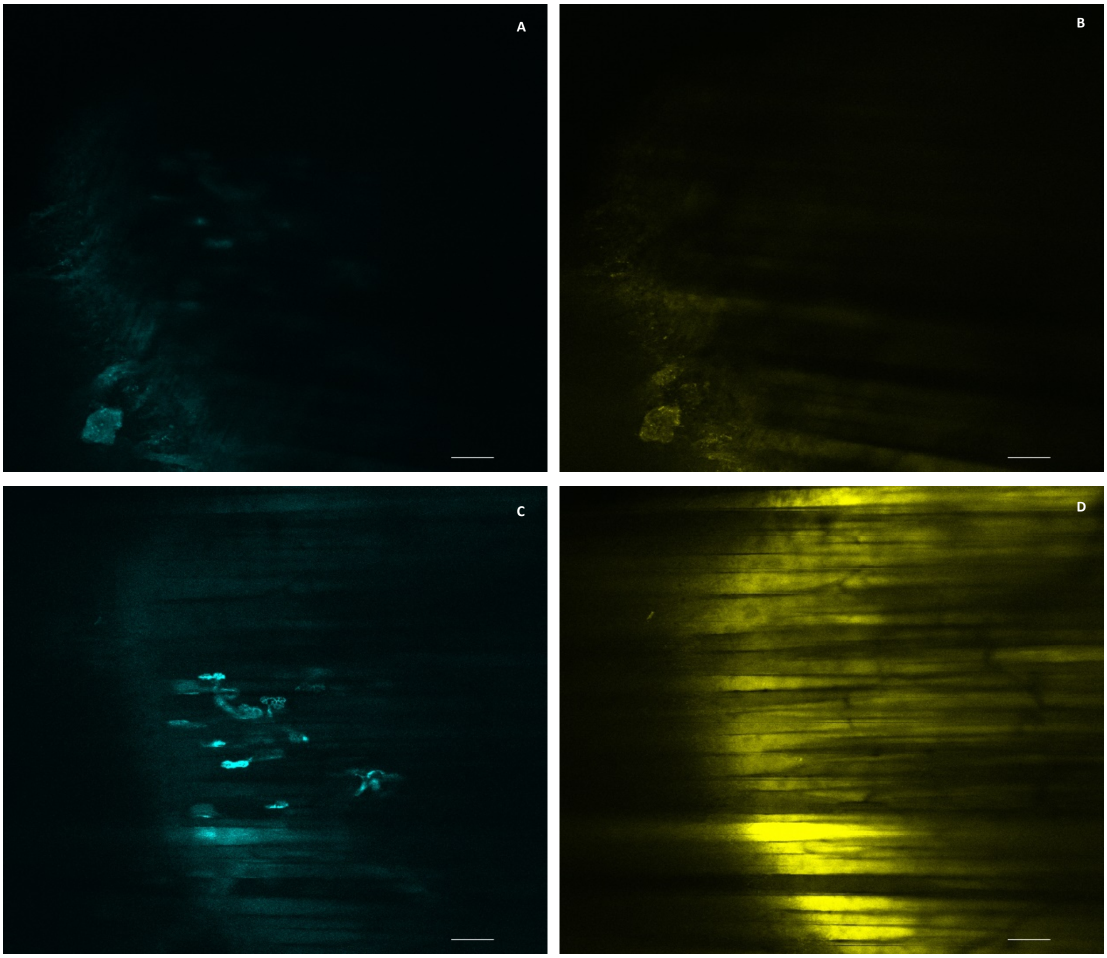

Figure S2. Video to illustrate the movement present in intra vital studies of the AT muscle

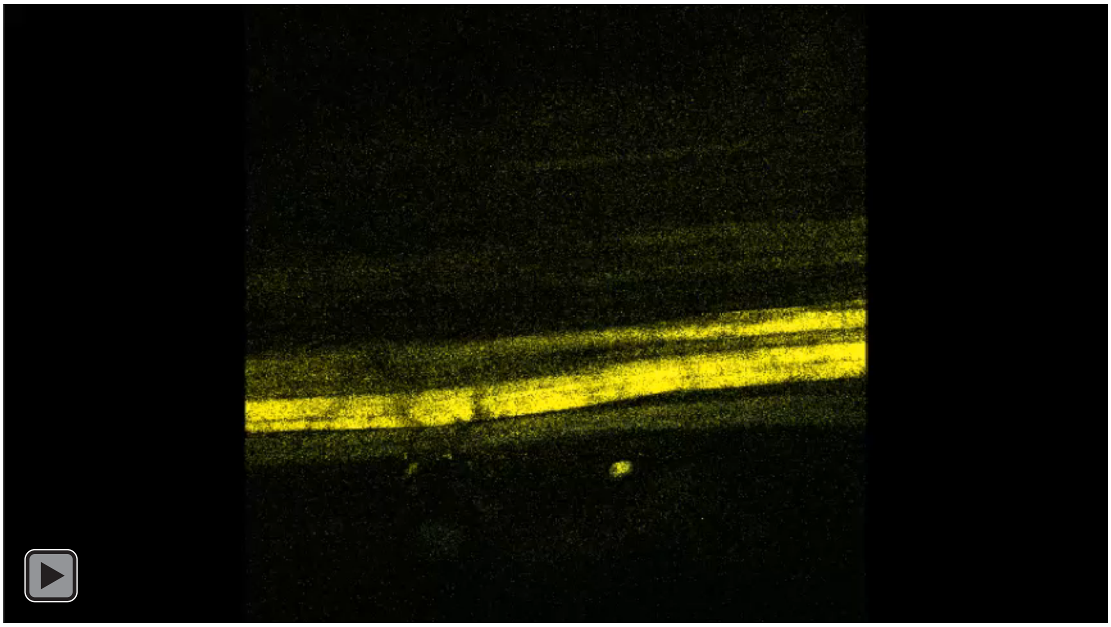

Time-lapse video (512*512 quality, every 10s captured)

Figure S3. Rate of amplex red oxidation by pe rmeabilised fibers (expressed as H_2O_2 generation) in the presence of specific ETC substrates and inhibitors prior to, and at 3, 7 and 21 days post-denervation and sham operated controls.

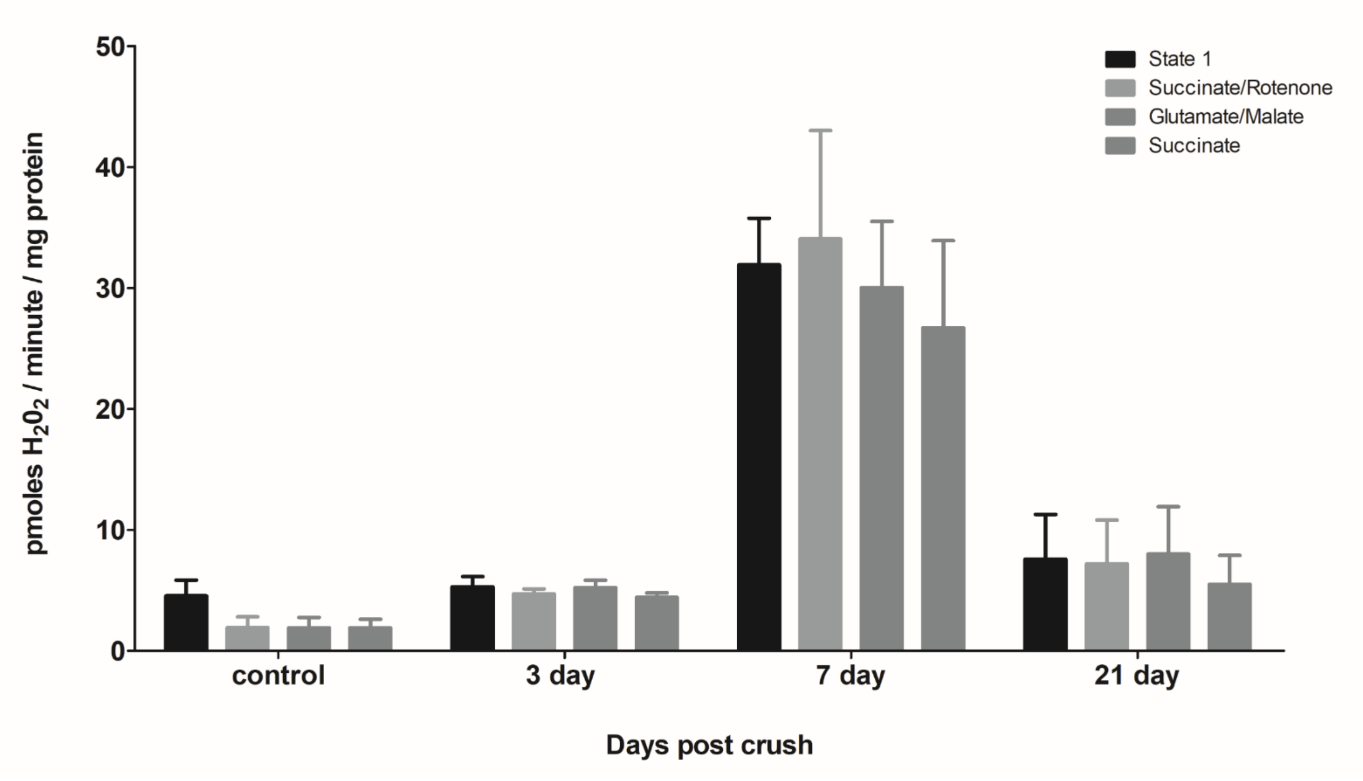

Supplementary Figure 3
